# Inaccuracy, Uncertainty and the Space-Time Permutation Scan Statistic

**DOI:** 10.1371/journal.pone.0052034

**Published:** 2013-02-07

**Authors:** Nicholas Malizia

**Affiliations:** GeoDa Center for Geospatial Analysis and Computation, School of Geographical Sciences and Urban Planning, Arizona State University, Tempe, Arizona, United States of America; University of Manchester, United Kingdom

## Abstract

The space-time permutation scan statistic (STPSS) is designed to identify hot (and cool) spots of space-time interaction within patterns of spatio-temporal events. While the method has been adopted widely in practice, there has been little consideration of the effect inaccurate and/or incomplete input data may have on its results. Given the pervasiveness of inaccuracy, uncertainty and incompleteness within spatio-temporal datasets and the popularity of the method, this issue warrants further investigation. Here, a series of simulation experiments using both synthetic and real-world data are carried out to better understand how deficiencies in the spatial and temporal accuracy as well as the completeness of the input data may affect results of the STPSS. The findings, while specific to the parameters employed here, reveal a surprising robustness of the method's results in the face of these deficiencies. As expected, the experiments illustrate that greater degradation of input data quality leads to greater variability in the results. Additionally, they show that weaker signals of space-time interaction are those most affected by the introduced deficiencies. However, in stark contrast to previous investigations into the impact of these input data problems on global tests of space-time interaction, this local metric is revealed to be only minimally affected by the degree of inaccuracy and incompleteness introduced in these experiments.

## Introduction

The space-time permutation scan statistic, introduced by [1], is used to identify clusters, or hotspots, of space-time interaction within patterns of spatio-temporal events. In certain contexts (e.g., when analyzing cases of disease or incidents of crime), such clusters are important to identify as they may indicate certain data generating processes or point to emergent trends [2]. A variety of metrics have been put forth to identify space-time interaction both globally (e.g. [3]–[6]) and locally (e.g. [7], [8]). The space-time permutation scan statistic (henceforth, STPSS) is among the latter and is most relevant for identifying such patterning when information pertaining to the distribution and dynamics of the underlying background population from which events are drawn is unavailable. The method has been utilized widely in practice, thanks, in part, to its implementation within the SaTScan software [9]. It has been employed to investigate spatio-temporal distributions of disease both prospectively [1], [10], [11] and retrospectively [12]–[20] and has also been used retrospectively to analyze distributions of wildlife sightings [21], [22], wildfires [23] and violent events [24], [25].

In spite of growing use of the STPSS, there has been no consideration of the impact inaccurate or uncertain input data may have on its results. This absence is troubling given the pervasiveness of such data deficiencies, especially in the context of geographic information [26]–[28] and a variety of studies which have demonstrated these deficiencies to have a concerning impact on the results of spatial [29]–[34] and spatio-temporal analyses [35], [36]. This study explores the possible consequences of deficiencies in the spatial and temporal accuracy as well as completeness of the input data on results of the STPSS. Specifically, this study endeavors to determine if a commonly encountered degree of these deficiencies is enough to prevent the method from successfully identifying hotspots of space-time interaction. Or, alternatively, from a practical perspective, will practitioners employing this method be misled by results affected by less than perfect input data?

A series of simulation experiments are employed in this pursuit, using both synthetic and real-world data. These experiments reveal the results of the STPSS to be relatively robust in the presence of the introduced inaccuracies. While the method is still affected by the deficiencies, their impact on results is less than expected based on the findings of previous research into the effect of such problems on global metrics of space-time interaction (i.e. [36]). The results of this work suggest the STPSS may be a versatile tool for investigations concerned with identifying local space-time interaction, even in the face of common data deficiencies. While these initial results are encouraging, it is important to restrain from overstating their worth. The scope of this work is limited and further investigations are needed before conclusions can be drawn about the ability of this method to handle such problems in a broader context.

The paper proceeds as follows. The introduction provides technical background on the STPSS as well as a brief overview of data quality deficiencies commonly encountered in spatio-temporal datasets. The methods section then describes the simulation experiments carried out as part of this study. Next the results of those experiments are reported while the final section discusses the findings and offers concluding remarks.

## Background

### Space-time permutation scan statistic

Part of a broader family of spatial and space-time scan statistics (see [7], [8], [37], [38]), the STPSS identifies the location and size of likely hotspots (or coolspots) of events in space and time and tests the significance of those concentrations using a Monte Carlo permutation approach. To calculate the statistic, the study area and time period of interest is first subdivided into areas (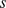) and time periods (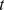) within which the observed number of events of interest is tallied. The total number of observed events (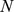) can be calculated as the sum of events observed in each of these areas across all times as shown in Equation 1.



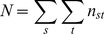
(1)The expected number of cases in each area and time period (i.e. 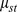) is calculated by conditioning on the observed marginals as shown in Equation 2. The STPSS assumes the function responsible for the generation of events operates uniformly across all time periods and areal subdivisions [1]. This is in contrast to other similar methods such as the cylindrical and flexibly shaped space-time scan statistics which assume spatial and temporal heterogeneity in the data generating process.



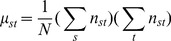
(2)Local concentrations of space-time interaction are identified using a cylindrical search window that moves methodically throughout the study area and time period of interest. The radius and height of the cylinder, which correspond to distances in space and time, respectively, vary as the cylinder moves across the study area and time period of interest. The number of events observed within the cylinder for all size/location/time combinations is compared to the number expected. The space-time permutation scan then maximizes the Poisson likelihood function described in Equation 3 across all cylinder radii, heights and starting locations to identify a most likely cluster (MLC) and possible secondary clusters. Pseudo-significance of the identified clusters is established using Monte Carlo hypothesis testing.



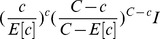
(3)In the likelihood function, 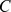 is the total count of cases, 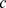 is the count of observed cases within the scanning cylinder, and 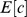 is expected number of observed cases within the cylinder based on the expectation of spatio-temporal randomness. Meanwhile, 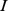 is an indicator function denoting a higher or lower than expected number of cases within the scanning window. When searching for areas of high concentration, this assumes a value of 1 when the cylinder has a greater number of cases than expected and 0 otherwise. The opposite is true when the method is employed to search for areas and times with a lower than expected number of cases (i.e. cool spots). Due to its inability to incorporate information on the dynamics of the background population, users must be aware that the method may erroneously identify clusters due to spatial and temporal variation in the underlying population from which events are drawn [1]. Where this is a potential problem and the necessary data are available, the more relevant cylindrical [7], [37] and flexible [8] space-time scans should be employed as they incorporate this knowledge directly.

As implemented in the SaTScan software [9], the results of the STPSS consist of a set of identified likely clusters and their associated parameters. For each cluster these parameters include the spatial coordinates of its center, its radius and temporal duration, a list of events included in the cluster, as well as the associated test statistic (generated using Equation 3) and a pseudo 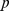-value. A most likely cluster (MLC) is identified as the cluster with the lowest pseudo 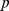-value. In addition, a series of possible secondary clusters are also identified.

### Data quality deficiencies

While the specific nature of any inaccuracies or uncertainties associated with the input data analyzed by the STPSS depends on the field of study in which it is applied, generally speaking, such problems are related to the geographic coordinates (i.e. the 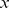 and 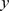 coordinates of events), their associated time stamps (i.e. 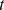) and the completeness of the dataset. Common problems encountered in spatio-temporal data include inaccurate or imprecise recording of the locations and times of events as well as under-reporting of the events. Additionally, uncertainty may result when the true locations and/or times of events are unknown and/or the completeness of the dataset under examination is questionable.

Individually and collectively, such deficiencies in the quality of input data have been shown to degrade the integrity of results for spatial and spatio-temporal analyses [29], [32]–[36], [39], [40]. However, the impact of such problems have not yet been investigated in the context of the SPTSS or any of the other space-time scan statistics. The sections below provide a brief overview of the existing literature on the problems associated with each of these characteristics of data quality as they pertain to spatial and spatio-temporal analyses. Specific attention is paid to problems pertaining to analyses in the contexts of health and crime. It should be noted that this review is based on the more extensive treatment of these topics provided by [36].

#### Spatial inaccuracies

Common sources of deficiencies in the location information associated with spatio-temporal event data include inaccurate geocoding, the application of privacy masks (i.e. aggregation to coarser scales or shuffling of locations), and uncertainty pertaining to latency and mobility [36]. The consequences and extent of these problems on spatial analyses are well documented and the relevant literature is discussed below. The effect of these problems on spatio-*temporal* analyses have been investigated to a far lesser degree; however, existing studies on this topic are covered here as well.

Inaccuracies in spatial event data due to the geocoding process (i.e. matching an address or other locational description to absolute geographic coordinates) are understood to be widespread in data created in this manner [41], [42]. The severity of the inaccuracies in geocoded data varies based on the quality of the underlying spatial data used in the geocoding process [43]–[48] as well as the density of addresses in the vicinity of the geocoded locations [49]–[52]. The detrimental impact of inaccurate geocoding on subsequent spatial analyses has been demonstrated by a number of studies. For example, [29] showed that geocoding errors affecting even a small number of observations (in their study, only 1% of the original data) impacted the results of analyses for local metrics of spatial autocorrelation. [32] observed variation in results of Kulldorff's spatial scan statistic, kernel density estimation and bivariate 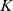 functions when different geocoding methods were employed to generate the raw data analyzed by the metrics. [33] demonstrated a decreased ability to recover relationships between environmental exposures and health outcome data as geocoding accuracy declined. [34] illustrated that moderate amounts of geocoding errors (affecting only 10% of records) were enough to modify disease distribution maps created using kernel density estimation. In a spatio-temporal context, [36] showed that a conservative degree of spatial inaccuracy in the form of simulated geocoding errors was capable of severely affecting the results of global tests of space-time interaction.

In addition to those introduced unintentionally via the geocoding process, spatial inaccuracies may also be introduced into spatial data intentionally to mask identity and preserve individual privacy [53]–[55]. Such inaccuracies are common in the context of health and crime data where the confidentiality of patients and victims (or offenders) is required. A common approach to the masking of locations is to aggregate the data to larger areal units [53], [54]. This approach, however, can yield different results than would be observed if the data were analyzed at the original level of spatial support [31], [35], [56]. Additionally, errors in the original spatial coordinates may result in the observations being aggregated to the wrong areal unit, further exacerbating such problems [52], [57], [58]. As an alternative to data aggregation, the privacy of individual events may be protected by assigning events to a new randomly generated location that falls within some specified radius of the original location [53], [59]. This perturbation approach has also been demonstrated to negatively affect the results of subsequent analyses in a manner proportional to the size of the radius [30].

#### Temporal inaccuracies

In spite of being equally relevant in terms of spatio-temporal analyses, inaccuracies in the temporal dimension of spatio-temporal data have received far less attention in the literature than their spatial counterparts. Such temporal inaccuracies encountered in event data commonly stem from the problems of latency and uncertainty.

The former is especially relevant to studies exploring the distribution of health and disease [60]. In this context, the period of time between an initial infection or exposure and the onset of symptoms or eventual diagnosis can, for certain diseases, be on the order of years or decades. However, most methods for analyzing spatio-temporal patterns (including the STPSS) require the specification of a single time (and place) where the event occurred, rather than incorporate the information available in a space-time path [61], [62] or employ an aoristic approach [63]. This, of course, relates to the discussion above on spatial inaccuracies, as during this time individuals may be mobile and it may be virtually impossible to assign a single discrete location to the disease case. This forced discretization in turn introduces errors into the analysis as the phenomenon cannot be accurately represented using a single point in space or time.

There is also the more general problem of uncertainty surrounding when an event that *can* be represented as a discrete event actually happened. A classic example, often offered, is that of a burglary event that occurs while the victim is away [64]. For all practical purposes, the burglary can be represented as a discrete event in space and time, however, given that the victim was away, it is often unknown *exactly* when the crime occurred. The the question remains: what should be used as the temporal coordinate of the burglary for analytical purposes? Should it correspond to the date and time the victim left and their home was untouched? Should it correspond to the date they discovered and reported the burglary? Or should it be some average of the two? This question is addressed by Ratcliffe's work on aoristic analysis [64], [65] who advocates that the entire time span should be used. This of course, is often not the approach employed in practical analyses. The only study (that this author is aware of) which explicitly investigates the consequences of this forced discretization in the context of spatio-temporal analysis is the aforementioned study by [36] which examined the effect of temporal uncertainty on tests of global space-time interaction. The study demonstrated that uncertainty in the temporal dimension of the input data can greatly distort the results of analyses, in some cases completely obscuring patterns of space-time interaction where they existed and in others creating them where they did not exist.

#### Incompleteness

In addition to the problems mentioned above concerning accuracy in the coordinates of recorded events, problems can also arise when the pattern of events recorded in the database is an incomplete representation of the pattern of interest in the real world. This mismatch is often a product of under-reporting of events or, in the case of geocoded data, incomplete geocoding.

Aside from the aforementioned positional error associated with geocoded data, the geocoding process may also fail to provide a set of spatial coordinates for an address. Such instances of “missed” geocodes are often the result of misspellings in addresses, an antiquated spatial reference file, the use of post office boxes rather than street addresses, or too stringent requirements on what constitutes an address match [66]. [44] examined the extent of missed geocodes for a variety of commercial geocoding vendors and reported match rates (i.e. successful geocodes) between 98% and 30%. Work by [39] suggests 85% as the minimum acceptable match rate, noting that below this rate maps created by aggregating individual events to census blocks are significantly different according to a Mann-Whitney 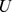 test. Researchers should exercise caution when working to increase the match rate however, as there is often a tradeoff between match rate and the positional accuracy of the geocoded points [41], [67].

Another source of missing data, often beyond the control of analysts, is under-reporting of events. This is a problem in a variety of applied contexts, especially epidemiology and criminology. In the former, the problem is mainly a result of under or misdiagnosing disease cases [60] while in the latter it results from under-reporting by both victims [68], [69] as well as police departments [70]–[72]. Certain types of crimes have higher rates of under-reporting. Sexual assaults, for example, have been noted to be among the least reported [68], [73].

## Methods

To explore the effect of these commonly encountered data deficiencies on the results of the STPSS, the experimental design employs two approaches. First, an experiment is undertaken in which a series of synthetic event patterns, exhibiting space-time interaction, are generated on a hypothetical landscape. These patterns are then perturbed to varying degrees by introducing spatial and temporal inaccuracies to the data and removing a percentage of events. The parameters associated with these perturbations are in line with what practitioners may encounter using real-world data and are based on estimates found in the existing literature or empirical observations. The effect of these perturbations on STPSS analyses are then assessed. The second approach, rather than relying on synthetic data, employs an observed pattern of criminal events for the analysis. The pattern of criminal events is perturbed in a manner similar to the simulated patterns above and the effect on the results of the STPSS is then assessed. The specifics of these different approaches are described in greater depth below.

### Synthetic data

For the first experiment, three synthetic patterns are generated on a hypothetical landscape. The study area measures 10 km square and the duration of the study period of interest is 100 days. Each of the original patterns generated within this space-time window include a background population of 200 events randomly distributed in space and time and two spatio-temporal hotspots: Cluster 1, in the northeast quadrant, late in the study period (seeded with 30 events) and Cluster 2, a smaller concentration in the southeast quadrant, early in the study period (seeded with 20 events).

**Figure 1:**
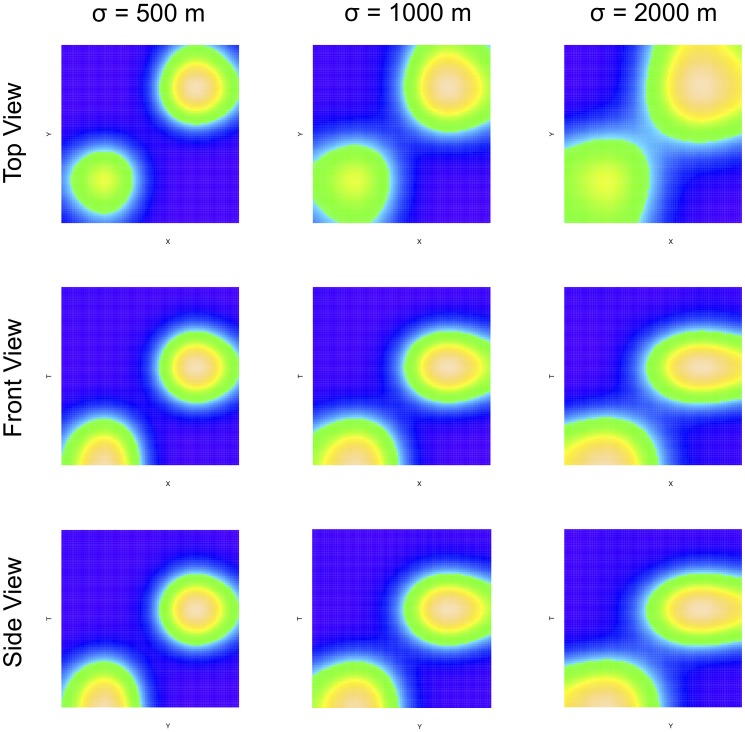
Intensity of the three simulated event patterns. Each panel shows a different perspective of a space-time cube for the three patterns. The left most column corresponds to the high intensity cluster pattern (where cluster events are concentrated in a smaller area) and the right-most column corresponds to the lowest intensity pattern. The top row shows an areal view of the space time cubes (i.e. a conventional map), the middle row shows a front view of the cubes, while the bottom row shows a side view. Lighter areas indicate a higher intensity of events.

The hotspots in the patterns are simulated independently of the background population by generating events surrounding two seed locations in space and time. The seed point for Cluster 1 is located at coordinates 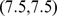 in space and at day 55 of the study period while the seed point for Cluster 2 is located at 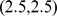 in space and day 10 of the study period. The events composing the clusters are generated by drawing coordinates randomly from normal distributions with a mean corresponding to the coordinates of the seed point in the respective dimension. The spatial intensity of the simulated space-time hotspots is varied in each of the three original patterns by adjusting the standard deviation associated with the distributions. The standard deviations used to generate the spatial coordinates for the events in hotspots of the three patterns are 500 m, 1,000 m, and 1,500 m, respectively. The standard deviation associated with the temporal dimension is held constant across all three patterns at 10 days. The intensities of events in space and time for the three patterns is shown in Figure 1. These different perspectives of the pattern illustrate the locations in space and time of the two simulated spatio-temporal hotspots. Based on these images, the change in the size, shape and intensity of the hotspots is apparent when the different values are employed for the spatial standard deviation. As this value (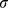) increases, the radius of the clusters increases. However, the associated height is maintained (because the temporal standard deviation remains the same) so they become more disc-like rather than spherical in shape. The simulated event patterns were then analyzed using the STPSS as implemented in SaTScan. For all the generated patterns, the scan identified Cluster 1 as the MLC and Cluster 2 as a secondary cluster with a highly significant 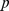-value. The specifics of these findings are discussed below in the results section.

**Figure 2:**
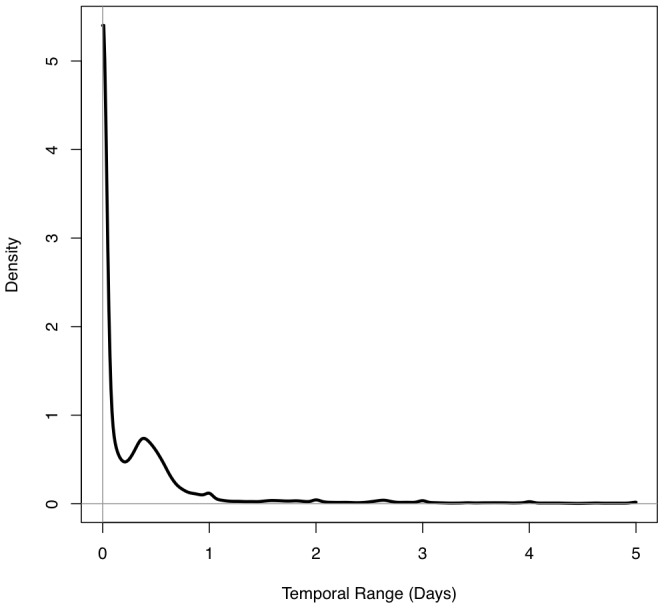
The distribution of temporal ranges within which burglaries and thefts are known to have occurred in Mesa, AZ for the period 2004–2009. Random draws from this distribution were used to offset the temporal coordinates of the original data.

With the original patterns simulated and analyzed, the accuracy of the datasets was then degraded based on quality estimates found in the literature (see [36], [50]). Three degrees of spatial inaccuracies were introduced into each of the datasets. These inaccuracies were introduced by randomly drawing an offset distance from exponential distributions with means of 50, 100, and 200 m (i.e [50]), corresponding to low, medium, and high levels of spatial perturbation designed to mimic empirically observed positional accuracy rates for geocoded data. The direction associated with the spatial offset was established using a random draw. Temporal inaccuracies were introduced by offsetting the temporal coordinates based on a random draw from an empirical distribution of suspected temporal inaccuracies for burglaries and thefts occurring in Mesa, Arizona. This distribution, composed of over 70,000 entries, was acquired from the Mesa Police Department. A kernel density estimation of the suspected ranges of inaccuracy is shown in Figure 2. To offset the temporal coordinates, a range is randomly selected from this empirical distribution. The range is then multiplied by a random value drawn from a uniform random distribution on the interval [−1,1] and the product is added to the original timestamp. The last step is taken to ensure that the resulting offset for the temporal coordinate occurs at a *random point* within the possible range, rather than consistently at the beginning or end of the possible period. Note also, with the abundance of zeroes in the distribution that not all of the temporal coordinates will be perturbed. Finally, the completeness of the pattern was then degraded by randomly removing 15% of the observations. Additionally, any events moved out of the study area or period during the perturbation process were omitted from subsequent analyses.

This methodology was used to create 1,000 degraded alternative versions for each of the original three patterns. The perturbed patterns were then individually analyzed using the STPSS in SaTScan. The results reported for the original patterns and the patterns of degraded quality are compared in the results section.

### Mesa, AZ burglary data

**Figure 3:**
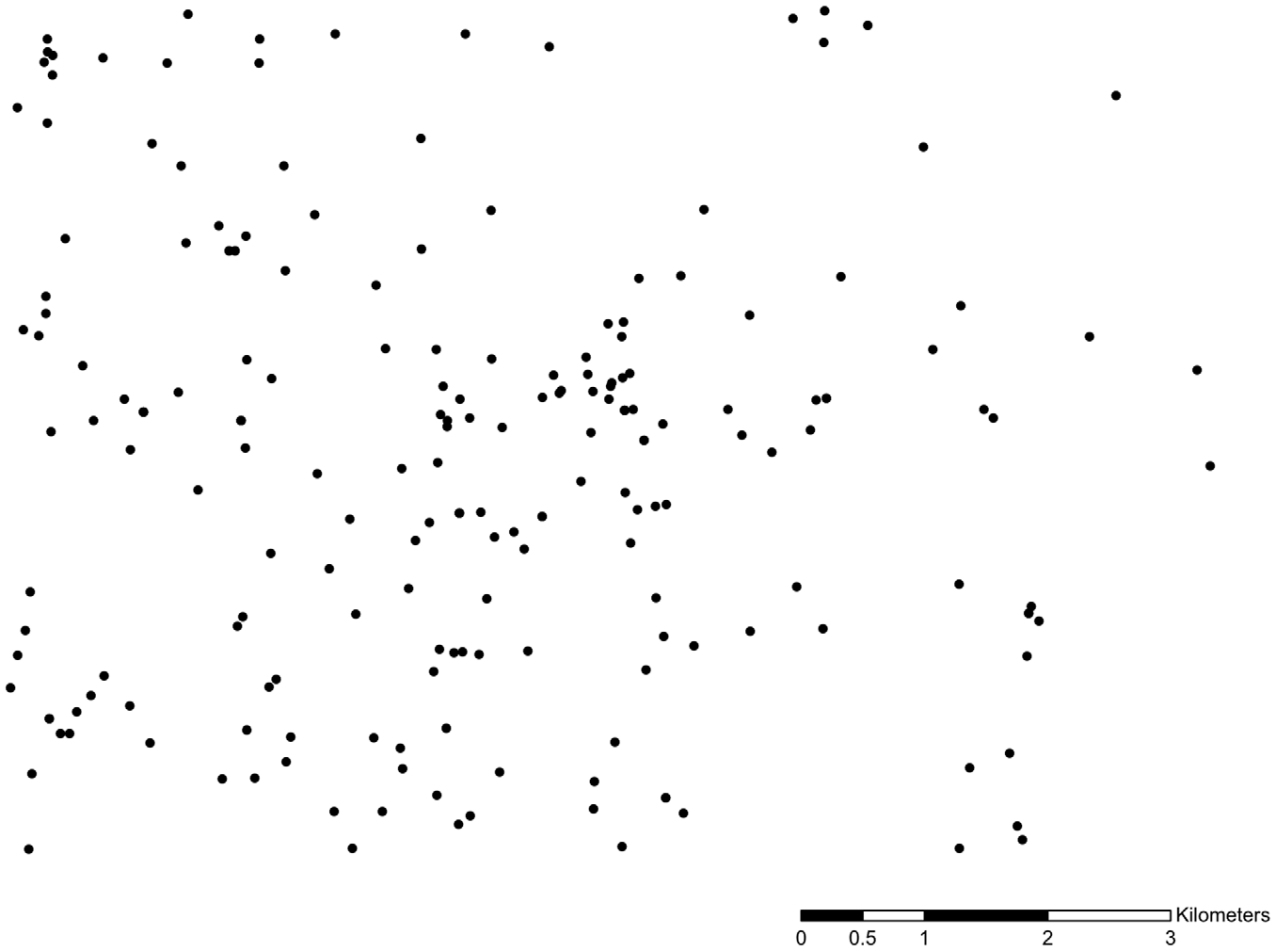
Sample of burglary events occurring in Mesa, Arizona during 2008 employed in the analysis. Additional geographic identifiers have been omitted from the map to preserve privacy.

Rather than rely solely on the synthetic data to explore the effect of data quality deficiencies on the STPSS, a second experiment was also carried out employing real-world data. Following a form similar to the one described above, this second experiment differs only in that it employs a pattern of burglary events observed in Mesa, Arizona during 2008 as the original event dataset for the experiment. The pattern is a sample of 200 burglaries drawn from the database kept by the Mesa Police Department. The raw data are shown in Figure 3. Spatial reference information has been omitted to preserve privacy. Again, the data were analyzed using SaTScan and the STPSS. The data were then perturbed in a manner similar to the synthetic data so that the spatial and temporal coordinates and the completeness of the data were affected. Given that these data are empirical, variability in the spatial intensity of the clusters was not used as a parameter in this experiment; however, the degree of perturbation was still varied as in the synthetic datasets. The results of analyses for the original and perturbed data are explored and compared in the following section.

## Results

The results from these experiments demonstrate the STPSS to be surprisingly robust to the moderate amount of perturbations introduced into the data. However, weak positive trends were observed indicating that more perturbation led to greater variability in results and greater likelihood of misidentifying the true MLC in the patterns. The experiments based on synthetic data also revealed a negative relationship between the effect of perturbation and the spatial intensity of clusters (i.e. less intense clusters were more affected by perturbations than their more intense counterparts). The results for both the synthetic and empirical data are explored in greater detail in the sections below.

### Synthetic data

**Figure 4:**
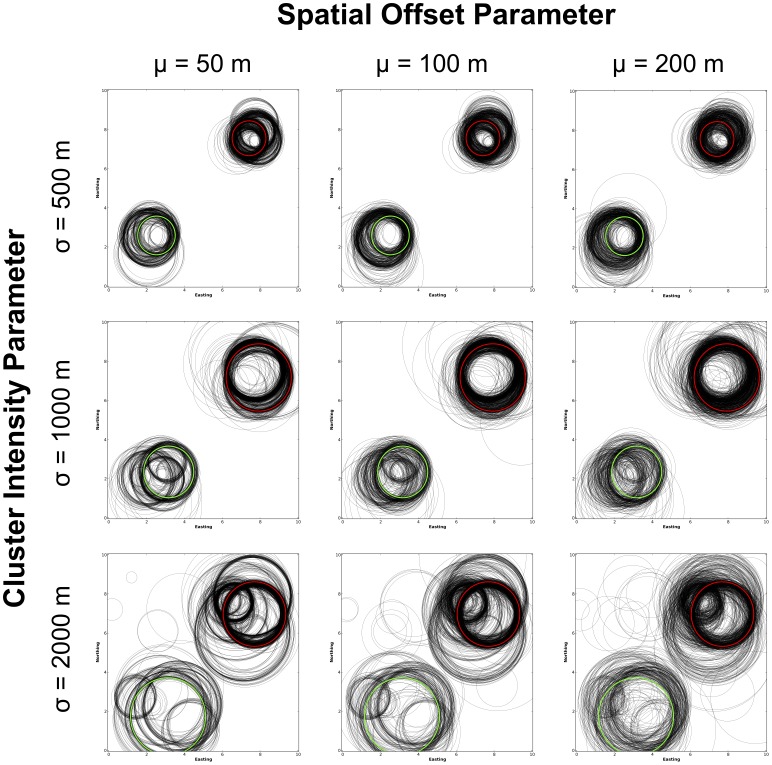
Plots of MLCs identified with the STPSS. The spatial footprint of the MLCs for the original datasets are shown in red and the secondary cluster with the next lowest 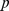-value is shown in green. MLCs from perturbed versions of the same dataset are shown in black. The intensity of the original clusters decreases from the top down while the intensity of perturbation increases from the left to the right. This layout is followed in subsequent graphics.

**Figure 5:**
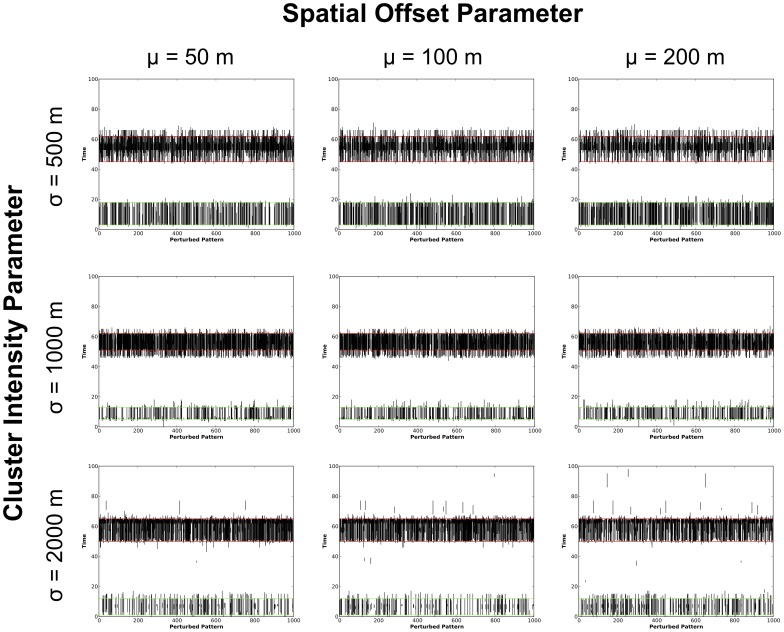
Plots of the duration of MLCs identified with the STPSS. The duration of the MLCs for the original datasets are denoted using horizontal red lines, secondary clusters are shown using green lines. MLCs from perturbed versions of the same dataset are shown as black vertical lines.

Figure 4 shows the locations of MLCs identified in the perturbed datasets and compares them to the locations of MLCs identified within their respective original patterns. Rows in the figure correspond to a different initial spatial intensity for the simulated hotspots. The top row shows the results for the patterns constructed using a standard deviation (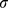) of 500 m, for the middle row 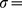
1,000 m and for the bottom 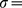
1,500 m. The columns, meanwhile, correspond to the different levels of spatial perturbation these original patterns were subjected to. The results in the left-most column are based on data whose spatial coordinates were perturbed based on a draw from an exponential distribution with a mean (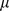) of 50 m, for the middle 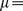
100 m and for the right 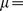
200 m. Note that the MLC (denoted as a red circle in the figures) identified for all three original patterns was in the vicinity of Cluster 1 in the northeast of the study area. A secondary cluster was also identified in all patterns by the STPSS (shown in green) in the vicinity of Cluster 2 in the southwest of the study area. These results are viewed from a temporal perspective in Figure 5. Here the duration of the MLC for each of the perturbed datasets are plotted as a vertical line. Horizontal red lines show the start and end times of the MLC identified for each observed dataset while horizontal green lines note the duration of secondary clusters. Again, the row and column structure mimics that of Figure 4 where the different rows and columns correspond to the various spatial intensity and perturbation parameters.

Together, the spatial and temporal perspectives of these results show that for the majority of the perturbed patterns, the STPSS identified Cluster 1 as the MLC in spite of the perturbations; although, Cluster 2 was also frequently identified as the MLC even though it was seeded with less events, thus having a larger initial 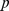-value (as can be seen in Table 1) and therefore a smaller likelihood of being identified as the MLC in the original data. Generally speaking, for the patterns where the clusters were more spatially concentrated (i.e. those where 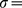
500 m or 1,000 m) the STPSS identified either Cluster 1 or 2 as the MLC. At these intensities, there were only a limited number of instances where MLCs unrelated to Clusters 1 and 2 were identified. Where the synthetic clusters were less spatially concentrated (i.e. where 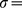
1,500 m) hotspots unrelated to Clusters 1 & 2 were identified as the MLC more frequently.

**Table 1:** Pseudo 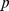-values as calculated by the STPSS associated with Clusters 1 and 2 for the hotspots of varying intensity.

Intensity (σ)	Cluster 1	Cluster 2
500 m	0.000037	0.00011
1000 m	0.00032	0.0025
1500 m	0.0036	0.063

**Figure 6:**
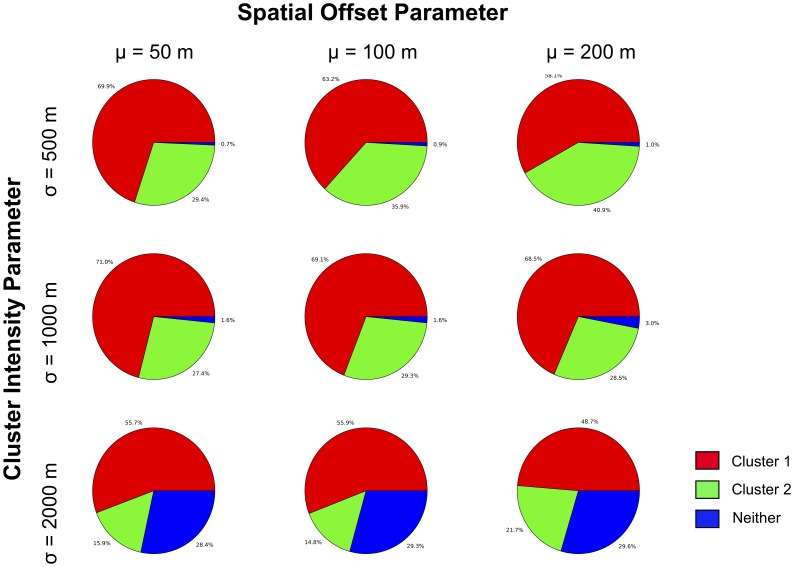
Pie charts showing the proportion of MLCs in the set of perturbed patterns located in the vicinity of Clusters 1 and 2.

**Figure 7:**
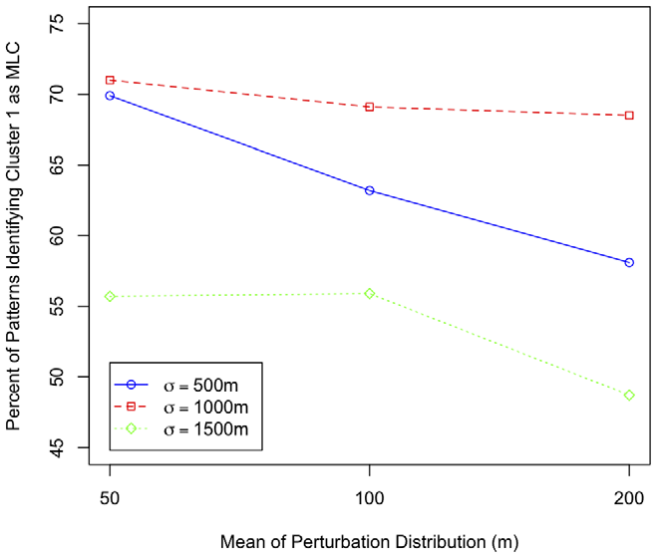
Percentage of patterns where Cluster 1 was reported as the MLC across the different intensity/perturbation combinations.

This is shown more clearly in Figure 6. The location of the MLCs identified in the perturbed data in relation to the location of Clusters 1 and 2 from the corresponding original datasets (i.e. those identified in red and green, respectively, in Figures 4 and 5) is explored further here. The collection of pie charts shows whether MLCs identified by the STPSS in the perturbed data are in the vicinity of Cluster 1, 2 or neither for the various combinations of original intensity and perturbation parameters. MLCs for the perturbed data were considered to be ‘in the vicinity’ of either Cluster 1 or 2 if their extent included the spatial and temporal center of the respective original cluster. The graphic shows that across all perturbation and intensity parameters, the majority of MLCs are identified in the vicinity of Clusters 1 and 2. However, a steady growth in the number of MLCs identified outside of these clusters is observed when the intensity of the original clusters is reduced. This appears unrelated to the level of perturbation introduced into the patterns. That being said, greater levels of spatial perturbation appeared to negatively affect the percentage of MLCs observed within the vicinity of Cluster 1 (the true MLC). With greater perturbation (i.e. an increase in the value of 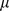, serving as a proxy for more spatial uncertainty) a smaller proportion of the MLCs for the perturbed datasets were identified in the vicinity of Cluster 1. This negative relationship is further illustrated in Figure 7. These results suggest that with decreased spatial accuracy, the STPSS may be less likely to pick out the true MLC amongst other possible clusters.

**Figure 8:**
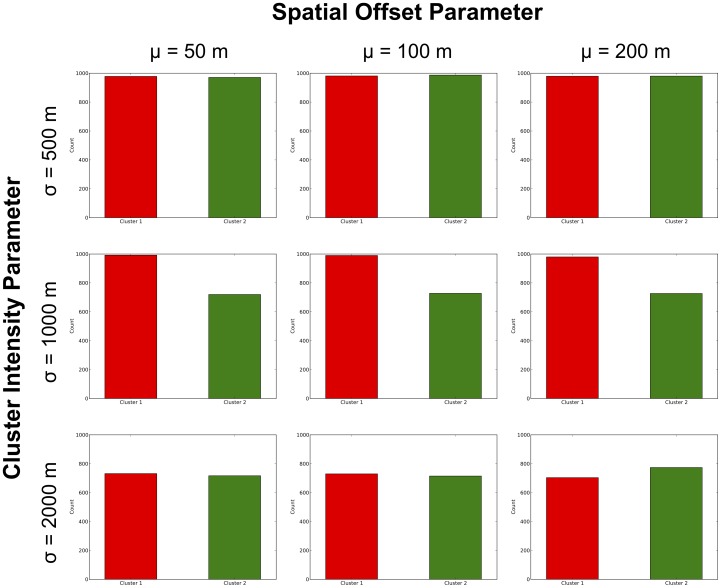
Number of perturbed patterns where Clusters 1(in red) and 2 (in green) were identified as “likely clusters” by the STPSS.

The results were further explored to determine how often likely clusters (not necessarily MLCs) were identified by the STPSS in the vicinity of the original Cluster 1 and 2. Again, a likely cluster is defined as being ‘in the vicinity’ of one of the original seeded clusters if it contains the spatial and temporal center of that original cluster. Figure 8 shows the count of perturbed patterns where the two clusters are identified as a likely cluster. For the perturbed patterns with original clusters of low to middling spatial intensity (i.e. 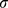 = 500 m or 1000 m) likely clusters are identified in the vicinity of Cluster 1 across almost all levels of spatial perturbation; however, this frequency drops off considerably when 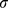 = 1500 m. Meanwhile, although likely clusters were consistently found in the vicinity of Cluster 2 across all levels of spatial perturbation when 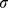 = 500 m, when the value for 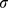 increased, likely clusters identified by the STPSS only identified Cluster 2 in 70–80% of the perturbed patterns. These results reiterate the findings from above that it appears less spatially intense patterns are more likely to be affected by the perturbations in the context of STPSS analyses.

**Figure 9:**
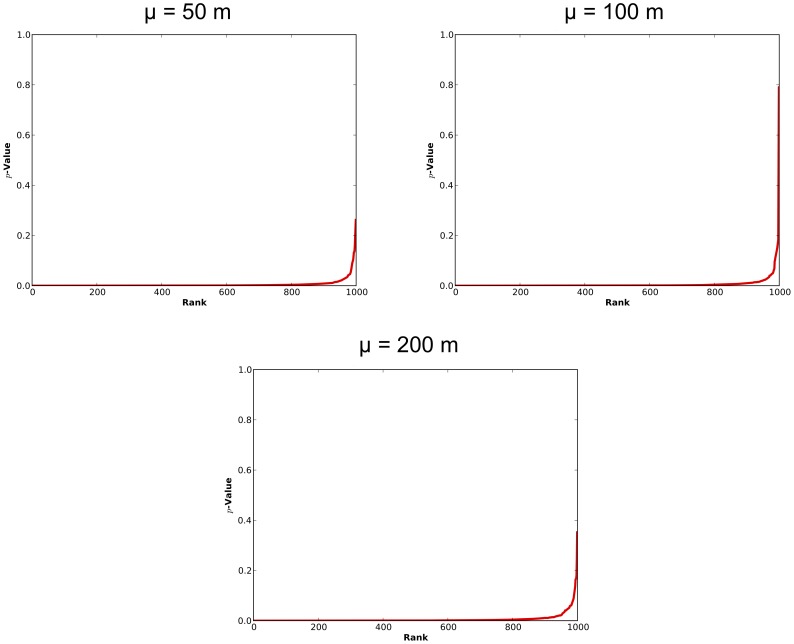
Pseudo 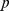-values determined by the STPSS for likely clusters identified in the vicinity of the original Cluster 1 (red solid line) and 2 (green dashed line) in each perturbed version of the original datasets.

This finding is corroborated when the 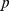-values associated with likely hotspots in the perturbed patterns identified in vicinity of the original Clusters 1 and 2 are examined. The 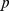-values for these likely clusters are shown in Figure 9 ranked from lowest to highest. Note that not all of the lines extend to the right-hand side of the figure, indicating that likely hotspots were not always identified in the vicinity of these clusters, mimicking the height of the bar in Figure 8. Of primary interest here though is the path of the lines, tracking the 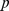-values for the identified clusters in each of the perturbed patterns. Where 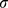 = 500 m, both lines (solid red and dashed green corresponding to the 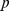-values for likely clusters identified in the vicinity of Clusters 1 and 2, respectively) follow the 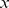 axis until the far right of the figure across all levels of perturbation. This indicates that across almost all of the perturbed patterns the clusters identified by the STPSS would be determined to be significant (if, for example, the 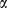 associated with the significance test were set at 0.05). Where 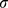 = 1000 m however, only Cluster 1 would be identified as being significant across most of the patterns. Cluster 2, aside from being identified by the STPSS as a likely cluster less often than Cluster 1 (i.e. the associated line does not extend entirely across the figure), also has larger 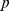-values associated with it. This trend is exacerbated where 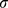 = 1500 m. Here, in only a small percentage of patterns is the 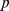-value for the hotspot identified in the vicinity of Cluster 2 significant. Cluster 1 is also affected, with less than half of the patterns reporting the presence of a significant hotspot. In contrast to the effect observed above on the identification of the MLC, there does not appear to be a relationship between level of spatial perturbation and the 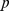-values for these identified clusters. Collectively, these results indicate that the STPSS results seem to be more vulnerable to perturbations when the initial spatial intensity of the examined pattern is weak to begin with. The level of perturbation, however, seems less important.

### Empirical data

**Figure 10:**
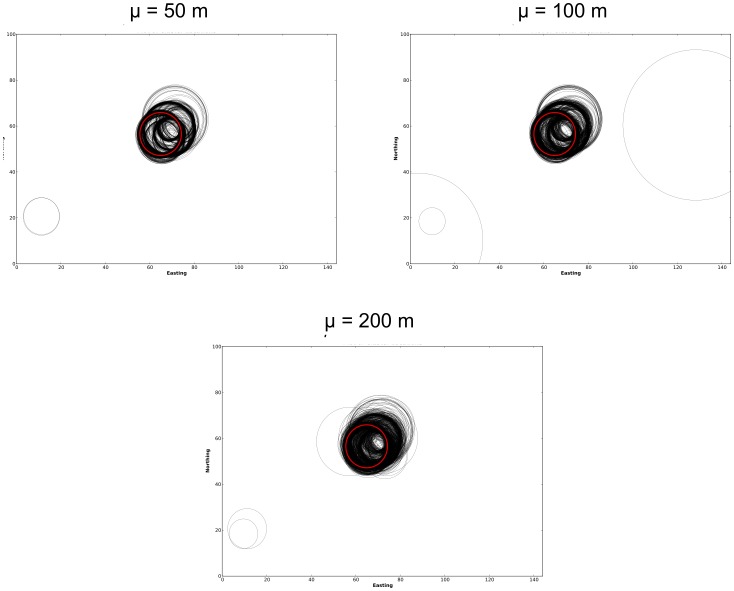
Plots of MLCs identified within the Mesa crime data using the STPSS. The spatial footprint of the MLC for the original dataset is shown in red. MLCs from perturbed versions of the same dataset are shown in black.

**Figure 11:**
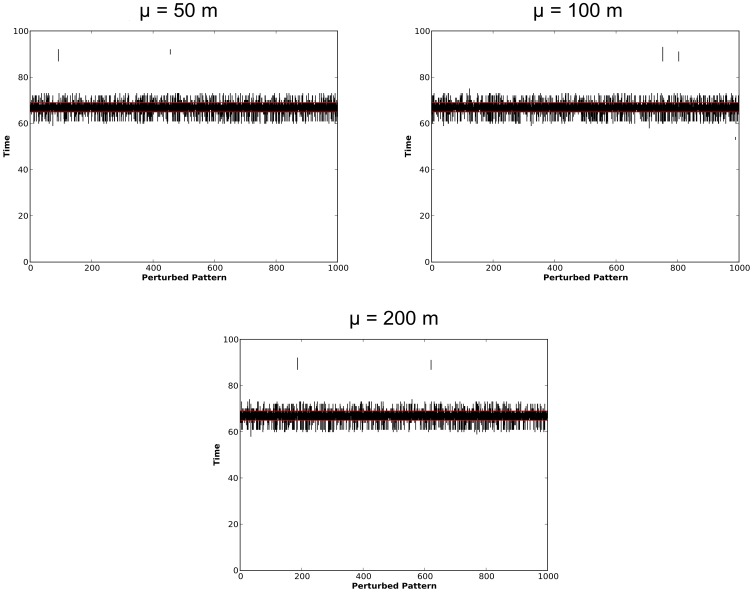
Plots of the duration of MLCs identified within the Mesa crime data using the STPSS. The duration of the MLC for the original dataset is denoted using horizontal red lines. MLCs from perturbed versions of the same dataset are shown as black vertical lines.

The results for the simulation experiments based on the Mesa crime data are now explored. Analysis of the original data using the STPSS revealed a single space-time hotspot within the dataset. As such, the impact of perturbations on clusters of different spatial intensities were not explored in this experiment. However, the effect of varying degrees of spatial perturbation and the effect of temporal inaccuracy and incompleteness on the detection of this hotspot were explored. First, the spatial and temporal distributions of the MLCs within the original and perturbed data are examined in Figure 10 and 11, respectively.

**Figure 12:**
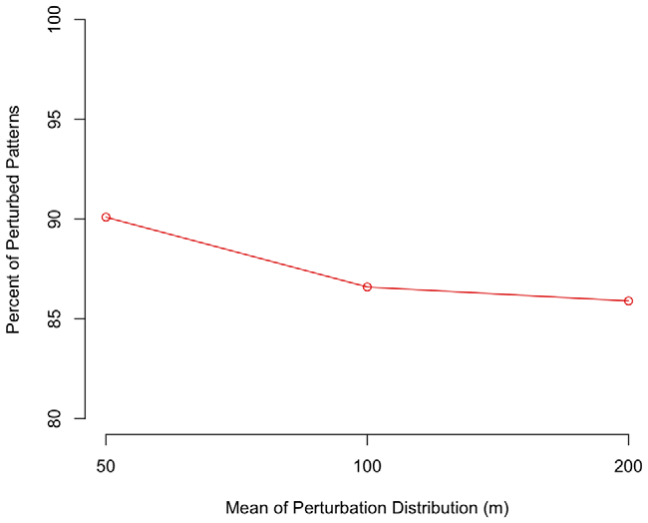
Percentage of patterns where the original MLC was reported as the MLC across the different perturbation levels.

The MLC for the original Mesa dataset are shown in red in Figures 10 and 11, no other statistically significant (at 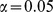) secondary clusters were identified. The MLCs identified within the perturbed versions of these datasets are shown on the same figures in black. As in the prior experiment based on the synthetic data, these initial explorations into the spatial and temporal distribution of the identified MLCs show stability in both dimensions across the various levels of spatial perturbation. Generally speaking, the MLCs identified within the perturbed datasets appear to be close to the MLC identified in the original dataset. This observation, however, is explored more formally in Figure 12. Here the percentage of MLCs in the perturbed data which are ‘in the vicinity’ of the MLC from the original dataset (in the formal sense defined above, i.e. include the spatial and temporal center of the original cluster) is tallied.

The figure shows that as spatial perturbation increases, there is a decreasing percentage of results for the perturbed patterns where the identified MLC includes the spatial and temporal center of the MLC identified within the original data. This trend was also observed within the results for synthetic data experiments as shown in Figure 7. These results indicate that as the level of perturbation increases, the STPSS is less likely to identify an MLC in its true location. While this may be the result of an overly stringent definition of ‘in the vicinity’, it does indicate greater variability in results with greater spatial perturbation.

**Figure 13:**
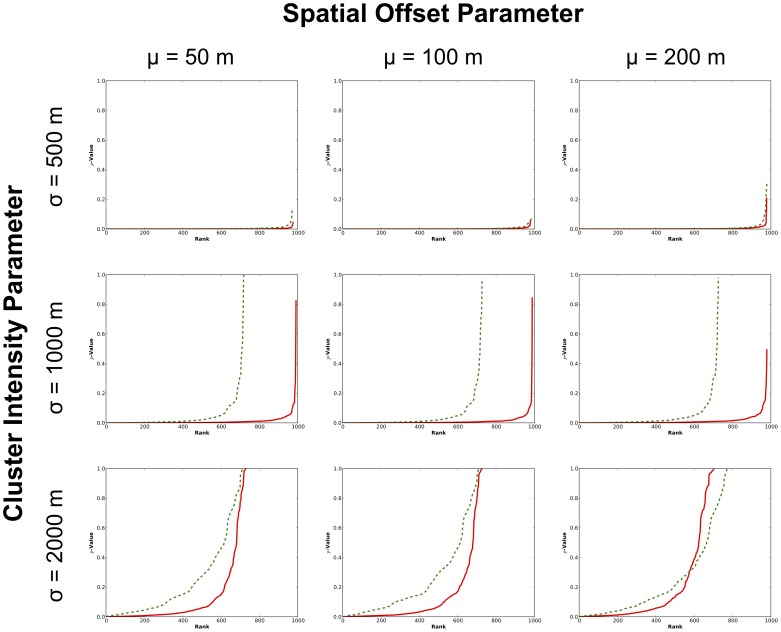
Pseudo 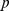-values determined by the STPSS for likely clusters identified in the vicinity of the original cluster 1 (red solid line) in each perturbed version of the original datasets.

Finally, the pseudo 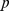-values associated with likely clusters identified in the perturbed datasets are explored in Figure 13. Specifically, the figure examines 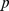-values associated with clusters located in the vicinity of the MLC from the original dataset. The 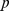-value associated with the MLC in the original pattern was observed to be 0.000063. Across the perturbed patterns, again, the likely clusters identified in the vicinity of the original MLC are also observed to be highly significant. Additionally, stability is observed across the various levels of perturbation: there appears to be no relationship between level of perturbation and 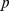-values.

## Discussion and Conclusion

While prior studies (i.e. [35], [36]) have shown global tests of space-time interaction to be highly volatile in the face of similar data deficiencies, collectively these findings demonstrate a marked departure from this precedent for this local method. Based on the experiments conducted here, the results of the STPSS appear to be quite robust to the moderate degree of the common data problems introduced. While there is an observed negative trend between degree of perturbation and ability to locate the correct MLC, especially within patterns with multiple significant hotspots, the relationship is weak at best, and not unexpected. What is surprising however, is how well the STPSS performs in the face of these common perturbations as compared to the global methods for detecting space-time interaction. [36] employed identical parameters to perturb data in his exploration of the effect of data inaccuracy on global tests of space-time interaction and found the results of those tests essentially devolved to randomness after perturbation. Similar findings were reported by [35] as well, although they only investigated problems associated with aggregating the original data in space. While this initial study offers a favorable view of the robustness of the STPSS, subsequent work will need to further explore this topic and these results in greater depth.

It should be noted that while all facets of uncertainty and inaccuracy discussed in the literature review were incorporated into the experiments here (i.e. the data were spatially and temporally perturbed and its completeness degraded) only in the case of the spatial perturbation was any sensitivity really explored. This is a consequence of two factors. First, it had already been shown that reducing the completeness beyond 85% of the original pattern can result in different results [39]. Given that 85% is the standard with which most geocoding is carried out, it provided a good baseline for the investigation carried out here. Second, in the case of the temporal dimension, changing the perturbation systematically (as in the case of the spatial perturbations) was not an option given the lack of research in this area on which to ground the sensitivity analysis. Further work is needed in this area to assess the accuracy of temporal coordinates in a variety of applied contexts.

While the results presented here cast a favorable light on the STPSS, care should be taken not to overstate their significance or overestimate the ability of this method to handle inaccuracies and uncertainty. The perturbations imposed on the data employed here were of a conservative nature. It is likely that far less favorable results would be observed if stronger degrees of inaccuracy, uncertainty and incompleteness were employed. Of particular concern may be the use of this method to identify patterns in cases of diseases with long latencies [60]. Additionally, the author cautions against the extension of these findings to other local tests of space-time interaction such as the cylindrical and flexible space-time scans as these have the added parameter of background population to account for. In the case of those methods, potential inaccuracy in accounting for spatially and temporally heterogeneous background populations offers an additional dimension of concern that may warrant further investigation.

In spite of these caveats, this research has shown that in contexts where researchers have reasonable confidence in the spatial and temporal accuracy and precision of their data they should also have confidence in the integrity of the reported results of the STPSS.
